# The role of metabolic reprogramming in kidney cancer

**DOI:** 10.3389/fonc.2024.1402351

**Published:** 2024-05-31

**Authors:** Ziyi Chen, Xiaohong Zhang

**Affiliations:** ^1^ The First Clinical College of Fujian Medical University, Fuzhou, China; ^2^ Department of Nephrology, Blood Purification Research Center, The First Affiliated Hospital, Fujian Medical University, Fuzhou, China; ^3^ Fujian Clinical Research Center for Metabolic Chronic Kidney Disease, The First Affiliated Hospital, Fujian Medical University, Fuzhou, China; ^4^ Department of Nephrology, National Regional Medical Center, Binhai Campus of the First Affiliated Hospital, Fujian Medical University, Fuzhou, China

**Keywords:** metabolism reprogramming, renal cancer, treatment, glucose metabolism, amino acid metabolism

## Abstract

Metabolic reprogramming is a cellular process in which cells modify their metabolic patterns to meet energy requirements, promote proliferation, and enhance resistance to external stressors. This process also introduces new functionalities to the cells. The ‘Warburg effect’ is a well-studied example of metabolic reprogramming observed during tumorigenesis. Recent studies have shown that kidney cells undergo various forms of metabolic reprogramming following injury. Moreover, metabolic reprogramming plays a crucial role in the progression, prognosis, and treatment of kidney cancer. This review offers a comprehensive examination of renal cancer, metabolic reprogramming, and its implications in kidney cancer. It also discusses recent advancements in the diagnosis and treatment of renal cancer.

## 1 Introduction

Metabolic reprogramming is a hallmark of malignancy first discovered a century ago. Reprogrammed metabolic activity has the potential to be utilized in the detection, surveillance, and management of cancer (1). Kidney cancer (KC) is predicted to be the 14th most common cancer globally by 2020, with 431,288 new cases reported, according to the Global Cancer Observatory (2). Despite the ongoing rise in incidence of KC, mortality estimates have reached a plateau (3). Significant roles are played by metabolic reprogramming in the prognosis and progression of kidney disease (4). By providing fresh perspectives on the diagnosis and treatment of metabolic reprogramming in renal cancer, this article examines the function of metabolic reprogramming in kidney cancer.

## 2 Metabolic reprogramming

In recent years, metabolic reprogramming has been defined as “changes in the bioenergetics of tumor cells” (5); ”some metabolic phenomena of cancer cell reprogramming (6)or “mechanisms by which cells reconnect their metabolism to promote proliferation and cell growth (7) “. The proposition is predicated on alterations in lactic acidosis and heightened glucose consumption in specific critical tumor regions (Warburg effect) (8). Primarily, cellular energy production (as ATP), amino acid synthesis, and the surrounding microenvironment are impacted by these modifications.

### 2.1 Metabolic reprogramming and tumors

Metabolic reprogramming is a phenomenon observed in malignant cells as they advance in development, adjusting their metabolic pathways. Mutations that lead to cancer formation allow nascent tumor cells to acquire metabolic traits that support cell survival, immune evasion, and rapid growth, making it a defining feature of cancer. This concept applies to classic oncogenes like MYC and KRAS, which can independently control cellular metabolism (1). Additionally, metabolic reprogramming influences the treatment of tumors. Given the distinct metabolic attributes exhibited by tumor cells in comparison to normal cells, it is possible to devise therapeutic approaches that specifically target the metabolic deficiencies of tumor cells. Additionally, one potential approach to treating tumors could involve manipulating the metabolic pathways of the cells containing the tumors. This could be achieved through specific metabolic inhibitors or by modifying the nutrient supply.

**Figure 1:**
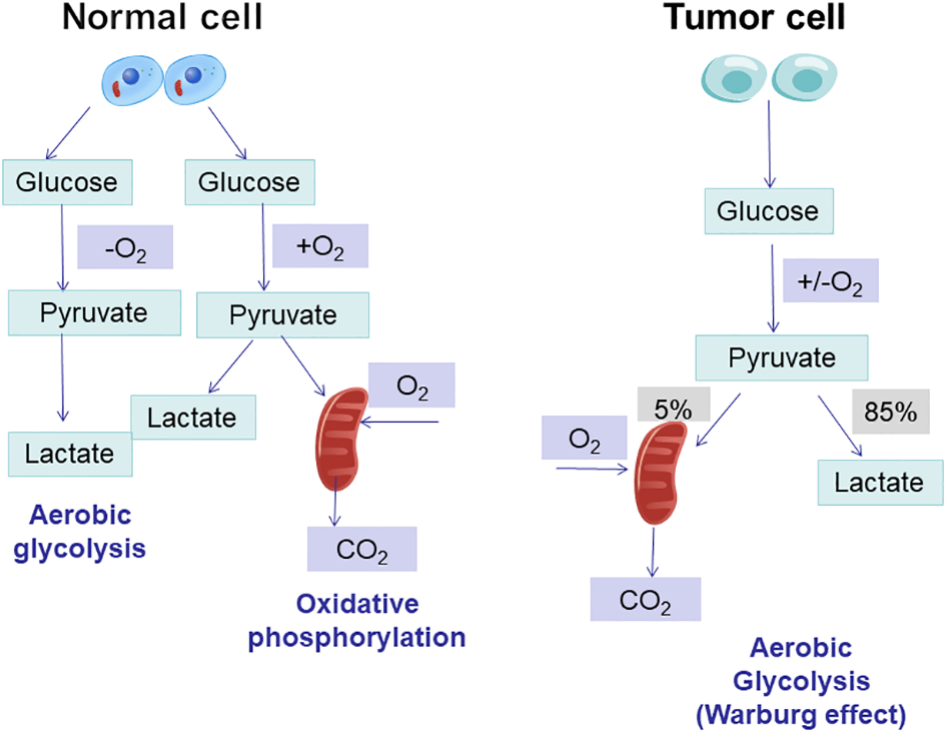
Regulation of glucose metabolism in cancer cells. Glucose metabolism in mitochondria mainly consists of glycolysis and the tricarboxylic acid cycle, and tumour cells enhance the conversion of glucose to lactate pathway through the glycolytic metabolic pathway.

Tumor cells generate adenosine triphosphate (ATP) via glycolysis, as opposed to the oxidative phosphorylation (OXPHOS) by which normal cells generate energy (5). Tumor cells exhibit a unique capability to consume large quantities of glucose for energy production through glycolysis, even in oxygen-rich conditions, known as the Warburg effect or aerobic glycolysis. Furthermore, metabolic reprogramming involves not only the Warburg effect but also various other metabolic alterations to adjust to different environmental conditions (4), including enhanced lipid synthesis, abnormal amino acid metabolism and altered lactate metabolism. Specific facets pertaining to metabolic reprogramming in tumors are as follows (**Figure 1**).

#### 2.1.1 Increased glycolysis

Normal cells typically metabolize glucose through oxidative phosphorylation in the presence of oxygen, resulting in the production of significant amounts of ATP. In contrast, tumor cells, even when provided with sufficient oxygen, tend to favor the glycolytic metabolic pathway, also known as ‘aerobic fermentation,’ to convert glucose into lactate. However, this conversion occurred at a reduced rate of OXPHOS. An excessive amount of lactate produced may cause the tumor microenvironment (TME) to become acidic. In addition to increasing the availability of ATP, lactic acid accumulation may also influence the ability of tumors to invade and metastasize (9). Proliferating cells prevent the accumulation of cytosolic NADPH and reduce ATP production by converting excess pyruvate to lactate. This promotes sustained cytosolic glucose metabolism and helps avoid feedback inhibition caused by ‘overproduction’ of mitochondrial ATP (10).

#### 2.1.2 Increased lipid synthesis

Glucose is the primary carbon source in most tumor microenvironments (TMEs) and is used for lipid synthesis through citrate. Conversely, cancer cells generate energy by oxidizing fatty acids in a lipid-rich TME. Tumor cells typically demonstrate increased resynthesis of fatty acids, redirecting energy production towards anabolic pathways that create phospholipids for cell membranes and signaling molecules (11). An abundance of evidence suggests that the lipid metabolism of immune cells and tumor cells in tumor microenvironments (TMEs) is essential for coordinating immunosuppression (9).

#### 2.1.3 Alterations in the metabolism of amino acids

Malignant cells often exhibit irregular amino acid metabolism patterns. For instance, specific tumors meet the metabolic needs of cancer cells by consuming a significant amount of glutamine. This process, known as glutamine anaplerosis or glutamine backfilling, leads to increased ammonia release. Exposure to ammonia can trigger autophagy in nearby cells, including cancer-associated fibroblasts (CAFs). Moreover, the activation of autophagy in CAFs by ammonia promotes the release of glutamine, which in turn supports the proliferation of tumor cells. Additionally, byproducts like aspartate and glutamate from glutamine metabolism play crucial roles in regulating tumor cell epigenetics, nucleotide synthesis, redox homeostasis, and overall metabolism (9). In addition to prostaglandin E2 (PGE2) and cyclooxygenase, the aforementioned pathways also involve adenosine signaling mechanisms. In the hours following tissue injury, adenosine concentrations in hypoxic tissues and TMEs increased significantly (12). Cell surface molecules CD73 and CD39 serve as nucleotide metabolizing enzymes, respectively. Adenosine synthesis is regulated by their conversion of ATP to AMP and AMP to adenosine, respectively (13). A correlation has been identified between heightened expression of CD39 and CD73 in tumors and an unfavorable prognosis in patients with non-small cell lung cancer, gastrointestinal cancer, and gynecological cancer (14). Cyclooxygenase 2 (COX2) overexpression is observed in a multitude of cancers (15). This overexpression is significantly associated with immunosuppression within the tumor microenvironment (TME) and substantial production of PGE2. Inhibiting the production of PGE2 and its associated signaling cascade has been shown to improve numerous components of the immune response against tumors, with colorectal cancer receiving the most attention (16).

The aforementioned attributes of metabolic reprogramming provide tumor cells with advantages in terms of proliferation and survival. Metabolic pathway modifications in tumor cells augment their resistance to the arduous microenvironment present within the tumor.

### 2.2 Metabolic reprogramming and immunity

The immune system consists of a variety of immune cells such as macrophages, neutrophils, monocytes, eosinophils, basophils, lymphocytes, and natural killer cells. While these cells are inactive during normal conditions, they quickly become activated and respond when exposed to infections, inflammation, or external triggers.

**Figure 2:**
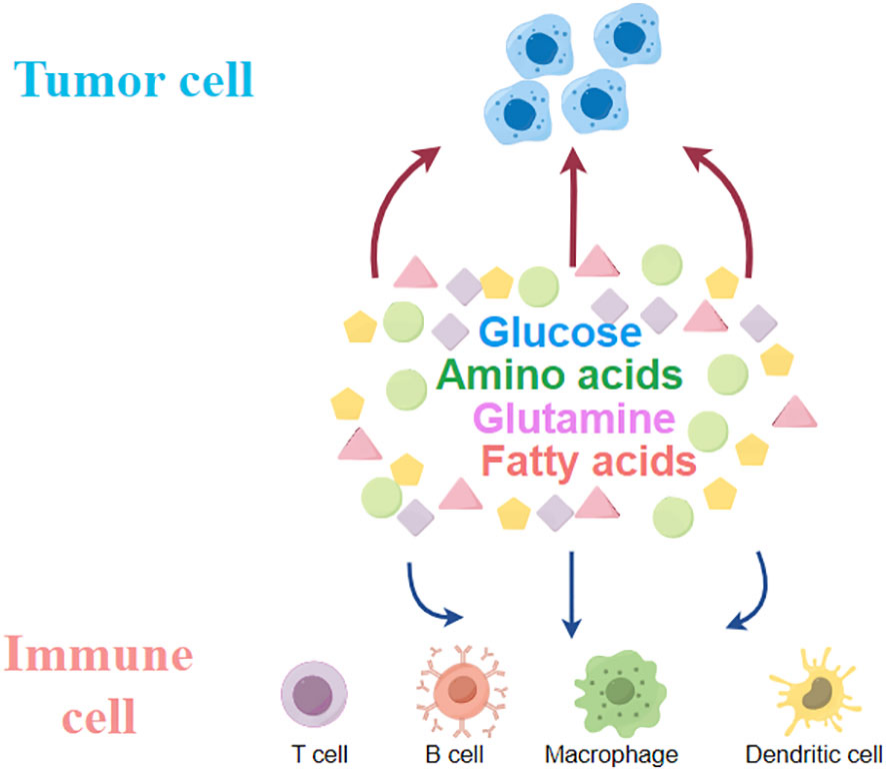
Tumour cells compete with immune cells within the tumour for glucose, glutamine, fatty acids and other amino acids.

T cells exhibit completely different metabolic patterns depending on their activation state (17). The metabolism of naïve T cells is essentially static, with zero proliferation, and therefore requires only minimal nutrient intake, minimal glycolysis rate and minimal biosynthesis to be maintained, and their ATP is mainly produced by OXPHOS (18). Once activated by an external stimulus to effector T cells (Teff), it exhibits a state of metabolic activation, increased nutrient uptake, increased rate of glycolysis, and accumulation of protein, lipid and nucleotide synthesis (19). At the same time, mitochondrial oxygen consumption is reduced, and eventually T cells gain the ability to grow and proliferate, generating progeny cells that perform effector killing functions (18).The metabolic pattern of memory T cells is similar to that of naïve T cells, maintaining a basic nutrient intake, a lower rate of glycolysis, and a dependence on OXPHOS to provide ATP (19). Enhanced glycolysis and mitochondrial metabolism are observed following B-lymphocyte activation induced by LPS or antigenic stimulation. It is worth noting that glycolysis serves as the primary metabolic pathway for activated B lymphocytes. In contrast, regulatory T cells (Treg cells) and M2 macrophages predominantly rely on oxidative phosphorylation (OXPHOS) generated through fatty acid oxidation (FAO) to meet their energy demands (20) (**Table 1** and **Figure 2**).

**Table 1:** Changes in energy metabolism in glycolysis, oxidative phosphorylation, fatty acid oxidation, and glutamine catabolism in different T cells.

Type	Changing of energy metabolism			
	Glycolysis	OXPHOS	FAO	Glutaminolysis
Naïve T cell	↓	↑	↑↑	↓
Treg cell	↓	↑	↑↑	↓
Teff cell	↑↑	↑		↓
Th1 cell	↑↑	↓	↓	↑
Memory T cell	↓	↑	↑↑	↓
Cytotoxic T cell	↑↑	↓	↓	↑

↓: decreased, ↑↑: Significantly increased, ↑: increased.

## 3 Metabolic reprogramming in renal cancer

Clear cell renal cell carcinoma (ccRCC), papillary renal cell carcinoma (pRCC), and smoky renal cell carcinoma (chRCC) are the three primary subtypes of renal cell carcinomas as determined by histological examination (21). Furthermore, renal cell carcinoma (RCC), collecting duct renal cell carcinoma, medullary renal cell carcinoma, and hereditary smooth muscle tumor disease are uncommon subtypes (21).CcRCC is the most prevalent subtype of RCC, comprising over 75% of all reported cases (21).Renal cell carcinoma (RCC) is sometimes referred to as a ‘metabolic disease’ due to the disruptions and alterations that occur in various metabolic pathways. Metabolic reprogramming in renal cancer is mainly triggered by the activation of the Ras-PI3K-AKT-mTOR pathway and the inactivation of the von Hippel-Lindau (VHL) gene (22). Myc and hypoxia-inducible factor (HIF) play vital roles in the metabolic reprogramming of renal cell carcinoma. This reprogramming affects glucose, fatty acid metabolism, and the TCA cycle across all RCC types. Furthermore, renal cancer involves the alteration of glutamine, tryptophan, and arginine metabolism to support tumor growth and tumorigenesis (21).

### 3.1 Reprogramming of metabolic genes

Essential genes involved in the regulation of metabolic reprogramming in renal cancer are VHL, PTEN, Akt, mTOR, TSC1/2 and Myc (22, 23). The tumor suppressor gene von Hippel-Lindau (VHL) is particularly important for ccRCC, and its frequent mutation or deletion causes dysregulation of several hypoxia-inducible factor (HIF) transcription factor families and their associated pro-oncogenic mediators (24, 25). Inactivation of VHL leads to activation of two VHL E3 ubiquitin ligase complex targets, HIF1α and HIF2α (encoded by HIF1A and EPAS1) (21, 24, 25). Under hypoxia in cancer cells, HIF1α and HIF2α are upregulated, and the transcription of several low-responsive genes involved in tumor growth, angiogenesis and metastasis, as well as genes related to glucose transport and metabolism, are transcribed (21, 26). HIF can drive the expression of several proteins and enzymes involved in glucose uptake and glycolysis, such as GLUT1 (glucose transporter-1), PGK (phosphoglycerate kinase), LDHA (lactate dehydrogenase), PDK1 (pyruvate dehydrogenase kinase) and HK (hexokinase) (27). HIF also inhibits the tricarboxylic acid cycle and oxidative phosphorylation (28).

Frequent mutations in Ras-PI3K-Akt-mTOR pathway genes (including PTEN, mTOR and PIK3CA) were also observed in RCC cells (29, 30). TCGA studies of ccRCC also detected mutations in several genes in the PI3K-AKT-mTOR pathway, PTEN, TSC1/2 and PIK3CA (31–33). TSC1 and TSC2 encode heparin and nodulin to form a complex that inhibits mTORC1 activation (34). Furthermore, inhibition of tumor suppressor 4EBP1 by mTORC1 enhances the expression of HIF-1 and HIF-2 (35, 36). Myc is a proto-oncogenic transcription factor, often overexpressed in renal cell carcinoma cells (37, 38), which plays an important role in reprogramming glutamine metabolism and fatty acid synthesis (37, 39).

### 3.2 Alterations in glucose metabolism

The presence of HIF in cancer cells is not correlated with the availability of oxygen (26). Increased expression of lactate dehydrogenase A (LDHA), the enzyme responsible for converting pyruvate to lactate, was observed in response to elevated levels of HIF (40). In healthy cells, glucose catabolism to lactate generates less energy than oxidative phosphorylation (41). Therefore, in order to meet the energy demands of cancer cells, they must consume a great deal of glucose. Elevated glucose transporter expression on the membranes of cancer cells is a contributing factor to elevated glucose consumption (42). The metabolic transformation referred to as aerobic glycolysis or the “Wartburg effect” is responsible for this (5). An increase in aerobic glycolysis expedites the provision of carbon intermediates required for the biosynthesis of amino acids, lipids, and nucleic acids (43). Conversely, monocarboxylic acid transporters (MCTs) remove lactate, the principal byproduct of glycolysis, from cancer cells in order to facilitate a positive glucose flux via glycolysis (44).

### 3.3 Alterations in the pentose phosphate pathway and the tricarboxylic acid cycle

The rate-limiting enzyme of the pentose phosphate (PPP) pathway, glucose-6-phosphate dehydrogenase (G6PD) is frequently upregulated in cancer cells (45).The pentose phosphate pathway, which is up-regulated, supplies ribose precursors to satisfy the high demand for 5-carbon sugars for nucleotide biosynthesis and to maintain intracellular redox homeostasis for growth and proliferation (46, 47). Reducing equivalents (NADPH) are utilized to impede oxidative stress. A concurrent elevation in lactate efflux fosters the development of an immunosuppressive microenvironment within the tumor.

HIF downregulates the tricarboxylic acid cycle in renal cancer cells by inhibiting metabolic fluxes to the TCA cycle through transcriptional activation of PDK1. This results in a decreased conversion of pyruvate to acetyl coenzyme A and the suppression of intermediates such as fumaric acid and β-ketoglutarate. Pyruvate carboxylase (PC) converts acetyl coenzyme A to oxaloacetate, which is the primary stable intermediate of the tricarboxylic acid cycle (48, 49). The neurotransmitter GABA, which is produced as a byproduct of glutamine metabolism, is converted to succinate, another tricarboxylic acid cycle intermediate, via γ-aminobutyric acid transaminase (48, 49). A constituent of the alpha-ketoglutarate dehydrogenase complex, dihydrolipoamide acetyltransferase controls the recycling of alpha-ketoglutarate (48). Experimental investigations have demonstrated that renal cell carcinoma cells exhibit a downregulation of these enzymes in comparison to normal renal cells (50).

### 3.4 Alterations in fatty acid metabolism

Renal cell carcinoma often associated with obesity (51).In renal cell carcinoma, lipid synthesis exceeds lipid degradation. Expression of enzymes involved in fatty acid oxidation is down-regulated in ccRCC cells compared to normal renal cells (52, 53). SCD1 is the enzyme responsible for lipid storage and is highly expressed in ccRCC (53). The β-oxidation pathway of lipids was down-regulated, however, the synthesis of carnitine, fatty acids, phospholipids and cholesterol were all expressed up-regulated in renal cell carcinoma (21). Higher levels of cholesteryl ester accumulation have been reported in the kidneys of patients with ccRCC. The accumulation of “lipid droplets” is considered a hallmark of clear cell renal cell carcinoma (ccRCC). Accumulation of lipid droplets near the endoplasmic reticulum (ER) contributes to the maintenance of ER integrity in ccRCC cells. Storage of lipid droplets is induced by the gene periplasmic protein 2 (PLIN2), which is upregulated in a HIF2-dependent pathway to maintain endoplasmic reticulum homeostasis and withstand cytotoxic stresses (54).

**Figure 3:**
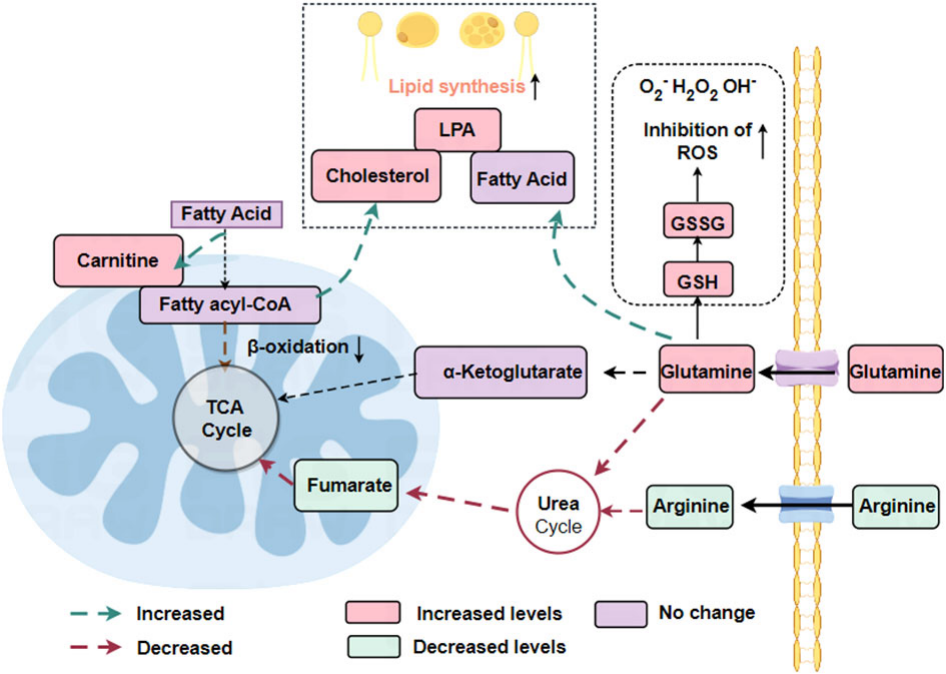
Reprogramming of fatty acid metabolism and glutamine metabolism in RCC. In renal cell carcinoma, where lipid synthesis predominantly exceeds lipid degradation, the β-oxidation pathway of lipids is down-regulated whereas the synthesis of carnitine, fatty acids, phospholipids, and cholesterol are all expressed up-regulated. The urea cycle is down-regulated, reducing the catabolism of amino acids such as arginine and glutamine.

Reprogramming of glycerophospholipid metabolism and arachidonic acid metabolism is characteristic of renal cancer (55). Glycerophospholipids are a source of phosphatidic acid (PA), lysophosphatidic acid (LPA) and triacylglycerol, which are forms of lipid storage (55). Arachidonic acid is an important derivative of membrane phospholipids, the synthesis of which involves a number of inflammatory enzymes such as lipoxygenases (LOXs) and cyclooxygenase-1 (COX-1) and cyclooxygenase-2 (COX-2) (55). Increased expression of the enzymes 5-LOX and 15-LOX2 and 15-hydroxyeicosatetraenoic acid, an immunosuppressive arachidonic acid, in renal cancer cells compared with normal renal cells (56). In RCC cells, LOX also promotes the secretion of the immunosuppressive chemokine CXCL2 and the cytokine IL10 and regulates immune escape from RCC cells (56). Also, the COX pathway of arachidonic acid metabolism is involved in the tumor-promoting pathway (57). Prostaglandin E2 (PGE2), a product of COX-2, promotes renal cell carcinoma invasion (57). COX-2 in renal cell carcinoma correlates with tumor size, stage and grade, suggesting that it may be a potential target in renal cancer cells (**Figure 3**).

### 3.5 Alterations in glutamine metabolism

Glutamate is an essential nutrient utilized by cancer cells for the maintenance of cellular bioenergetics and biomass. Additionally, it is a constituent of both protein and lipid synthesis (21). Glutamine acts as a precursor for the synthesis of glutathione (GSH), an antioxidant that operates within cells, as well as a metabolic intermediate in the form of α-ketoglutarate, which plays an indirect role in the TCA cycle. The conversion of glutamine to α-ketoglutarate is facilitated by the enzyme glutaminase (GLS) (58).

Metabolomic analysis of postoperatively resected tissues from patients diagnosed with clear cell renal cell carcinoma (ccRCC) showed a significant rise in glutamine utilization and uptake by tumor tissues compared to normal paired renal tissues. Additionally, an elevated glutathione content in tumor tissues was positively correlated with the clinical progression of ccRCC patients, including tumor stage and prognosis (21). GSH, an amino acid tripeptide consisting of glutamate, cysteine, and glycine, is present in numerous prokaryotic cells and nearly all eukaryotic cells (22). An important metabolic process involving glutamine is the metabolism of GSH. In the human body, the main forms of GSH are oxidized glutathione (GSSG) and reduced glutathione. Reduced GSH acts as an intracellular antioxidant and is transformed into GSSG under the influence of glutathione peroxidase. The balance between glutathione and oxidized glutathione (GSH/GSSG) is tightly regulated in renal cell carcinoma (21). Glutamine regulates redox processes in cancer cells via GSH as well. As a ROS substrate, glutathione is oxidized to GSSG to reduce ROS levels. This indicates that glutamine and glutathione act as the cell’s internal antioxidant system to maintain the survival of healthy tumor cells. The oncogene c-Myc can upregulate glutaminase expression, impacting the glutamine metabolism of cancer cells (59). The conversion of glutamine to glutamate is catalyzed by glutaminase. Inhibiting glutaminase or depriving ccRCC cells of glutamine in the culture medium reduces cell survival, revealing a dependency on exogenous glutamine. This highlights the importance of exogenous glutamine and GLS in the proliferation of tumor cells.

Furthermore, renal cancer cells exhibit a downregulation of the urea cycle, which impedes the catabolism of glutamine and arginine, among other amino acids (21). Increased tryptophan metabolism via the xanthine (KN) pathway leads to enhanced immunosuppression. By reprogramming metabolic pathways, energy (ATP) and other molecules necessary for cell proliferation (lipids, phospholipids, and ribose) are produced, allowing renal cancer cells to evade the immune system and withstand hypoxia, nutrient depletion, and oxidative stress (**Table 2**).

**Table 2:** Findings related to glutamine metabolism in renal tumor cells.

Year of study publication	metabolic mechanism	research object	The main findings of the study	reference
2011	Upregulation of free fatty acids in renal cell carcinoma	Patient-derived renal cancer cells	Elevated glutamine levels in kidney cancer	(60)
2015/2016	Increased expression of glutathione peroxidase 1 (GPX1) in ccRCC cells; Inhibition of glutamine-depleted enzyme expression via the GSH/GSSG pathway	138 matched pairs of clear cell ccRCC and normal tissue/RCC cells	Glutamine maintains cellular redox homeostasis by scavenging ROS; high-grade, high-stage and metastatic ccRCC are associated with elevated glutamine levels and the GSH/GSSG pathway	(48, 49)
2019	HSP60 silencing activated the MEK/ERK/c-Myc pathway to enhance glutamine-directed metabolism	Clear cell renal cell carcinoma 786-O and 769-P cell lines	Low expression of HSP60 enhances cell growth in ccRCC	(61)
2019	Macrophage-secreted IL-23 enhanced Treg functions in glutamine addicted tumors	ccRCC patients tumors from a Shanghai cohort and ccRCC tumor data from The Cancer Genome Atlas (TCGA) cohort; fresh human ccRCC tumors and murine tumor cells	IL-23 is a promising target for immunotherapy in ccRCC	(62)
2023	PHF8 is recruited by c-MYC to the promoter regions of TEA domain transcription factor 1 (TEAD1) to transcriptionally up-regulate TEAD1 then TEAD1 up-regulates GLUL transcriptionally	786-O cells (VHL-null cells)	PHF8-GLUL axis plays an essential role in ccRCC tumor growth and lipid depositionPHF8-GLUL	(63)

## 4 Metabolic reprogramming in the treatment of ccRCC

ccRCC is frequently associated with mutations in genes that cause hypoxic alterations, the most common of which is VHL (64). VHL mutations lead to the accumulation of HIF-a in cells, which in turn upregulates the expression of vascular endothelial growth factors (VEGFs) (65). Prior to this, the mainstay of treatment for ccRCC was the use of VEGF receptors (VEGFR) or inhibitors such as sunitinib leading to regeneration of the target vessel (66), but inhibitors have limited efficacy and can cause many adverse effects such as vascular toxicity and off-target effects (67). Based on metabolic reprogramming, in ccRCC, we can follow the therapeutic approach of hepatocellular carcinoma and use glycolysis inhibitors to suppress tumor cells (68), while early clinical studies have also demonstrated that targeting the glycolytic pathway can effectively inhibit cancer progression (69).

### 4.1 HIF-2a inhibitors

HIF-2a is a key downstream effector protein of the VHL tumor suppressor protein, which is frequently mutated in ccRCC (70), and promotes tumorigenesis and metastasis by regulating angiogenesis, cell proliferation and metabolism; therefore targeting the HIF-2a pathway could be used to treat ccRCC (71). First-generation drug PT2399 shows superior activity to sunitinib and is effective against sunitinib-resistant tumors (71) (NCT02293980). Second-generation drugs such as PT2977 (MK- 6482, Belzutivan) can overcome some of the limitations of first-generation compounds (72).

### 4.2 FAS inhibitors

Upregulation of FAS expression in ccRCC increases fatty acid levels and provides energy for cancer cells and post-translationally modified proteins (71). Preclinical experiments show that the FAS inhibitor C75 inhibits invasiveness and proliferation of ccRCC (73). TVB-2640 is a novel FAS inhibitor that demonstrated promising clinical activity and safety in a phase I clinical trial (74) (NCT02293980).

### 4.3 Glutaminase inhibitors

Glutamine is essential for energy production, redox stability maintenance, and macromolecule synthesis in cancer cells (71). In colorectal cancer, glutamine-like substance (GLS) functions as a compensatory mechanism to partially stimulate cell proliferation and restore the tricarboxylic acid cycle (75). CB-839, a GLS inhibitor, has demonstrated encouraging outcomes in preclinical investigations and augments antitumor functionality in animal models when combined with everolimus, a frequently utilized mTOR inhibitor for the treatment of ccRCC (71).

### 4.4 IDO inhibitors

IDO is an enzyme involved in the role of tryptophan catabolism via the renal urinary alkaline pathway (71). IDO promotes tumor metastasis by depleting tryptophan and activating T cells, inhibiting immunosuppression in the local tumor microenvironment and suppressing anti-tumor T cells. Thus, IDO has emerged as a potential therapeutic target for cancer. Epacaostat, a selective IDO-targeting inhibitor, showed promising results in preclinical trials by improving lysis of tumor antigen-specific T cells; however, side effects such as toxicity and lack of efficacy were identified in clinical trials (76). Some IDO inhibitors, such as KHK2455, LY3381916 and MK-7162, are undergoing clinical trials to assess their safety, tolerability and antitumor activity (77).

### 4.5 Reduction of arginine

In ccRCC, the use of the polyethylene glycol form of arginine deaminase (ADI-PEG20) can limit tumor growth by reducing circulating levels of arginine by catabolizing it to citrulline, however this treatment may be limited by ASS1 re-expression (75).Clinical trials have demonstrated the safety, tolerability and clinical efficacy of ADI-PEG20 in reversing drug resistance in patients with arginine dystrophy tumors (78).

This article focuses on the anoxic processes involved in glucose metabolism in cancer cells, specifically highlighting the pentose phosphate pathway and tricarboxylic acid cycle. It also emphasizes the connection between renal cell carcinoma and obesity. Additionally, it provides detailed descriptions of the specific changes in enzymes related to the fatty acid oxidation pathway and outlines the developmental course of glutamate metabolism in renal carcinoma. However, this article does not delve into the relevant metabolic pathways for tryptophan and arginine, only mentioning them in relation to specific treatment protocols. Another article (75) also discusses kidney cancer-related genes, fatty acids, glucose metabolism, tricarboxylic acid cycle, glutamic acid metabolism, and specific processes. The literature proposes using radionuclide imaging for diagnosing renal cell carcinoma. Furthermore, in this paper new drugs such as KHK2455, LY3381916 and MK-7162 are suggested for treating IDO inhibitors. Second-generation drugs like PT2977 (MK-6482) or Belzutivan are proposed as HIF-2α antagonists that can overcome certain defects of first-generation drugs.

## 5 Conclusion and future directions

Metabolomics studies have provided a number of small molecules that may be used to diagnose and predict kidney cancer, and which hold promise as biomarkers of kidney cancer. However, these interpretations are limited to mapping identified metabolites to pathways, while many important features remain undefined. Subsequent experimental work is required to demonstrate causal inferences arising from genomic analyses. The dynamic nature of the metabolome means that it may be difficult to identify the direction of protein/metabolite ←→ disease.

By analyzing indications such as metabolites, subgroups of patients with similar metabolic characteristics can be more accurately identified. This contributes to a deeper understanding of the heterogeneity of the disease and provides a basis for personalized treatment. Moreover, therapeutic strategies targeting metabolic vulnerability are based on targeted interventions to the weakness of specific metabolic pathways or links.

In the future, we can improve our understanding of disease progression at the individual level by integrating biological data from multiple genomics and combining a multilevel approach to observe the biological effects of different therapeutic pathways, which will ultimately improve the cure rate and reduce the mortality rate of renal cancer through other approaches such as targeted therapies.

## Author contributions

ZC: Writing – original draft, Writing – review & editing. XZ: Funding acquisition, Supervision, Writing – review & editing.
